# Supplementary material for: Industrial source identification of polyhalogenated carbazoles and preliminary assessment of their global emissions
Source: Nat Commun. 2023 Jun 22;14:3740. doi: 10.1038/s41467-023-39491-5 (PMC10287696; doi:10.1038/s41467-023-39491-5)
Supplement: Supplementary file 1 — Supplementary Information [file 41467_2023_39491_MOESM1_ESM.pdf]

Supplementary Information for

Industrial source identification of polyhalogenated carbazoles and preliminary assessment of their global emissions

Yuxiang Sun [1,2,3], Lili Yang [2,3], Minghui Zheng [1,2,3], Roland Weber [4], Jerzy Falandysz [5], Gerhard Lammel [6,7], Chenyan Zhao [1,2,3], Changzhi Chen [1,2,3], Qiuting Yang [2,3], Guorui Liu [1,2,3,*]

[1] School of Environment, Hangzhou Institute for Advanced Study, UCAS, Hangzhou 310024, China.

[2] State Key Laboratory of Environmental Chemistry and Ecotoxicology, Research Center for Eco-Environmental Sciences, Chinese Academy of Sciences, Beijing 100085, China.

[3] College of Resource and Environment, University of Chinese Academy of Sciences, Beijing 100049, China.

[4] POPs Environmental Consulting, Lindenfirststr. 23, 73527 Schwäbisch Gmünd, Germany

[5] Medical University of Lodz, Faculty of Pharmacy, Department of Toxicology, Muszyńskiego 1, 90-151 Łódź, Poland

[6] Max Planck Institute for Chemistry, Mainz, 55128 Germany

[7] RECETOX, Faculty of Science, Masaryk University, 60177 Brno, Czech Republic

[*] Corresponding author. E-mail: grliu@rcees.ac.cn (G. Liu)

This PDF file includes:

Supplementary Method 1 to 3

Supplementary Figure 1 to 5

Supplementary Table 1 to 6

Supplementary Figure 1

Structure of polyhalogenated carbazoles with numbering of substitution positions. (X indicates halogen atoms, and m and n indicate their number: 1 ≤ m + n ≤ 8).

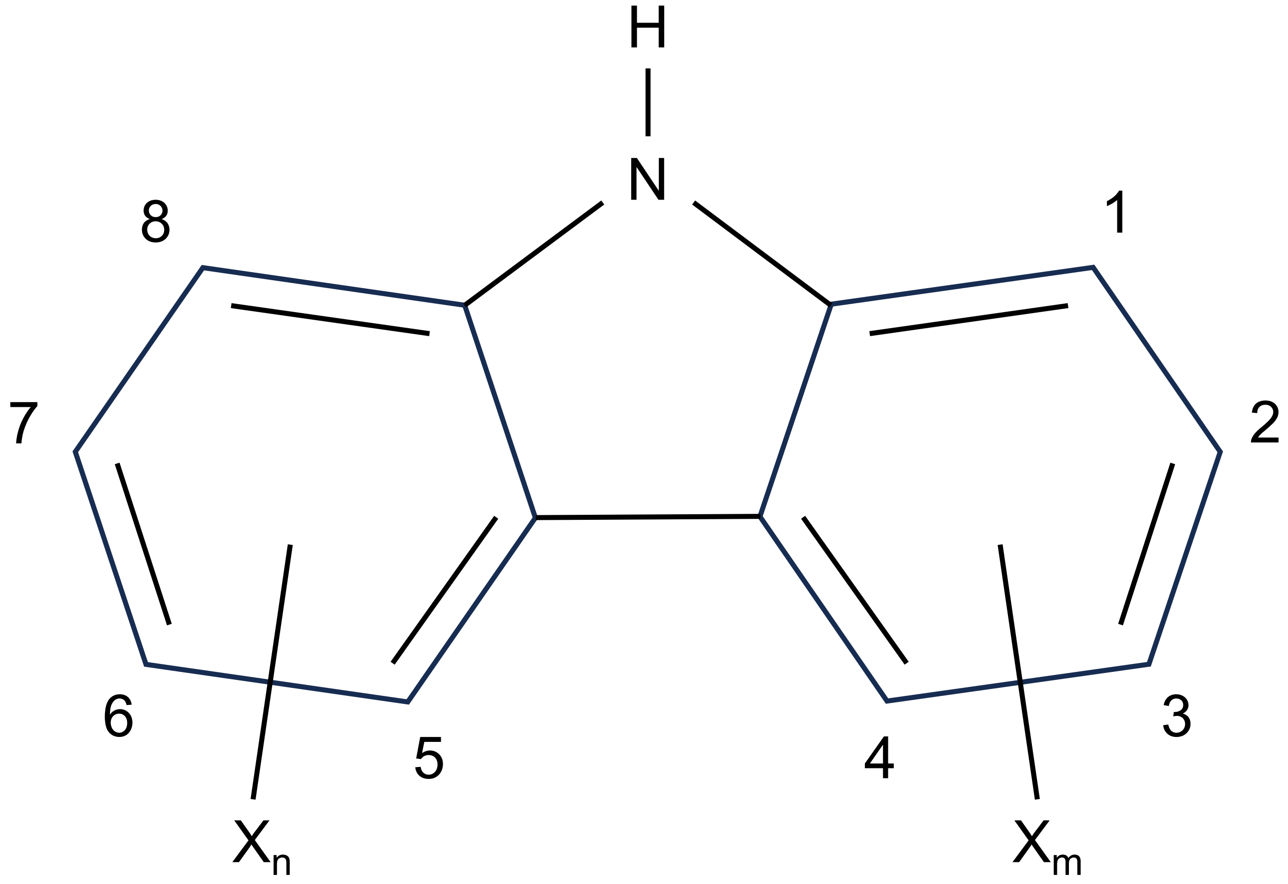

Supplementary Figure 2

Environmental occurrences of polyhalogenated carbazoles cited in this research[1-11]. Map sourced from Database of Global Administrative Areas free vector data.

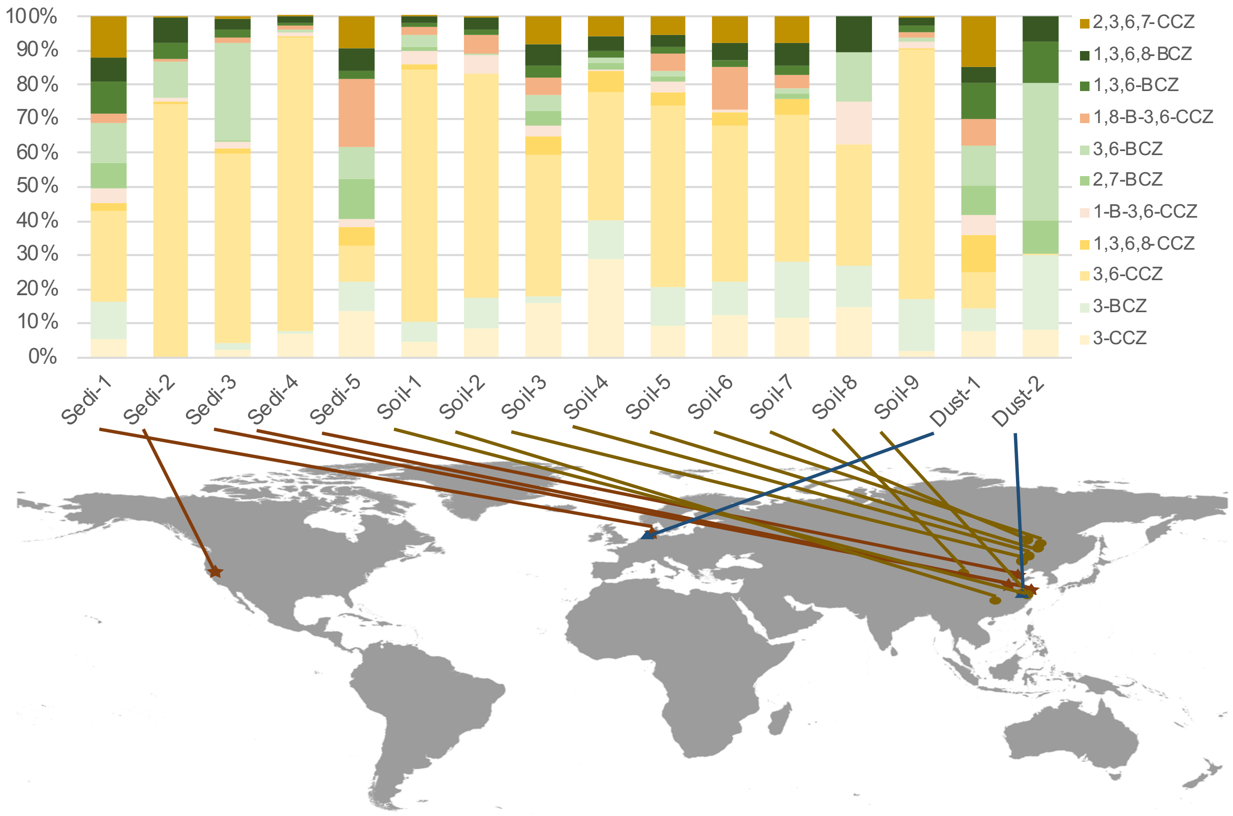

Environmental occurrences and references	Location
Sedi-1[1]	Northern Germany
Sedi-2[2]	San Francisco Bay, USA
Sedi-3[3]	Lake Tai, China
Sedi-4[4]	Zhou Shan, China
Sedi-5[5]	Qingdao, China
Soil-1[4]	Taizhou, China
Soil-2[6]	Zhoucheng, China
Soil-3[7]	Northeast Provinces, China
Soil-4[7]	Northeast Provinces, China
Soil-5[7]	Northeast Provinces, China
Soil-6[7]	Northeast Provinces, China
Soil-7[7]	Northeast Provinces, China
Soil-8[8]	Tibetan Plateau, China
Soil-9[9]	Hangzhou, China
Dust-1[10]	Derenda, Teltow, Germany
Dust-2[11]	Hangzhou, China

Supplementary Figure 3

Concentrations of polyhalogenated carbazoles (PHCZs) in sediment from Yaer Lake, an area suffering historical pollution from discharge of organic chemical production. In 1976, discharge of the chlor-alkali chemical plant led to the accumulation of polycyclic aromatic hydrocarbons, dioxins, and organochlorine pesticides in lake silt. Dredging work was carried out in 2002 for treatment, and silt was deposited in the soil pile area. By comparing the concentrations of PHCZs in different areas with those in ponds 1–5, a higher concentration was obtained in sediment from the pile soil area, which was formed during the desilting project in 2002. The results indicate that wastewater is an important discharge path of PHCZs in chemical production.

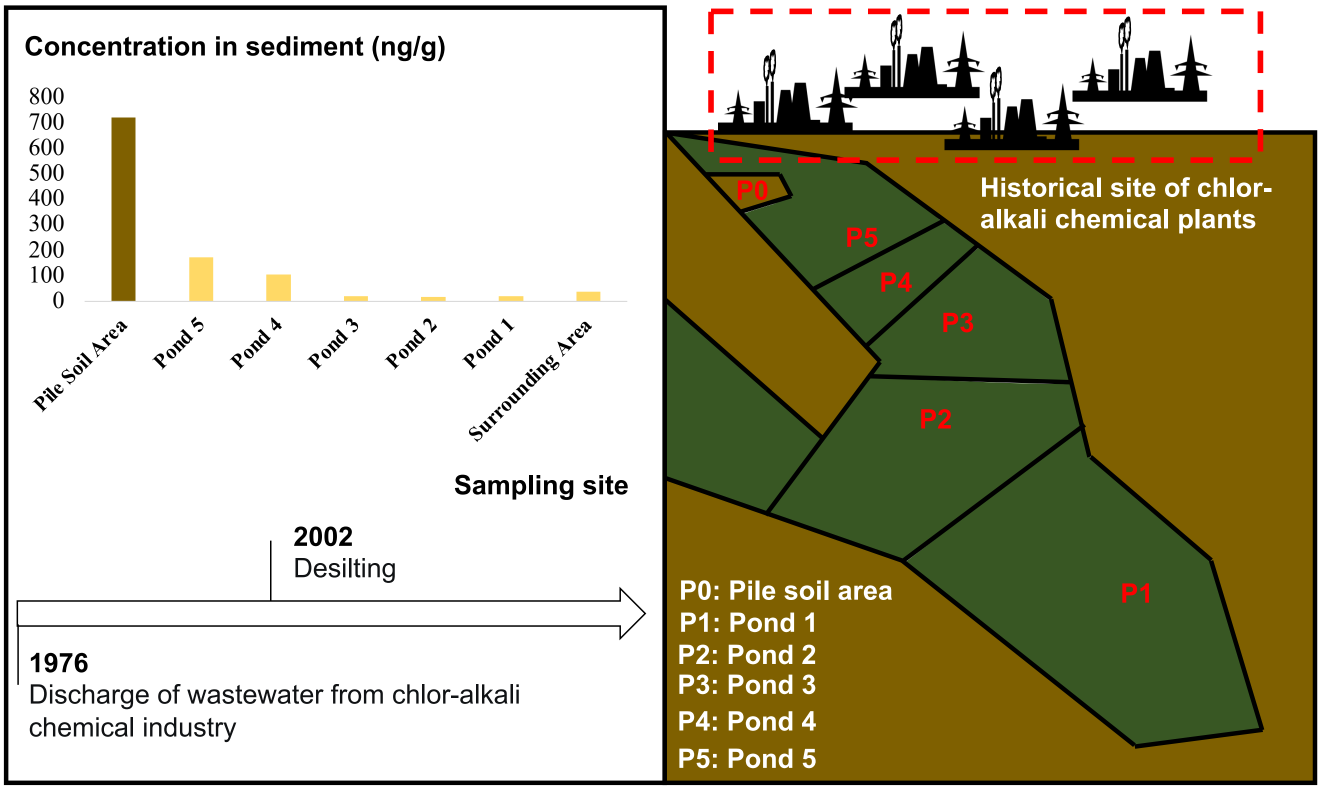

Supplementary Figure 4

Production of (a) Coking[12], (b) iron ore sintering[13], (c) electric arc furnace steelmaking[13] and (d) incineration amount of municipal solid waste[14] in major countries and regions. Map sourced from Database of Global Administrative Areas free vector data.

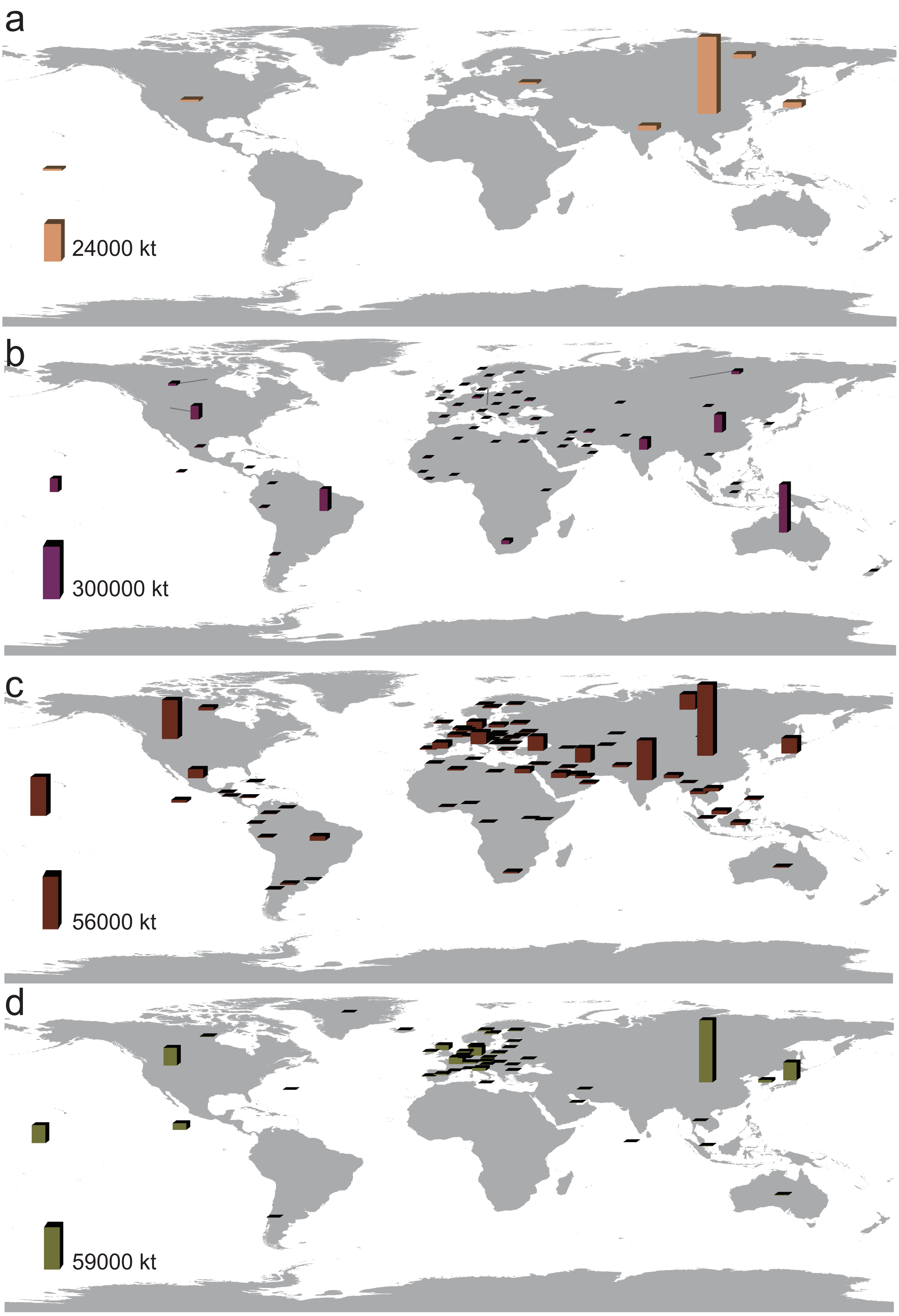

Supplementary Figure 5

Scanning electron microscopy of industrial fine particulate matter samples

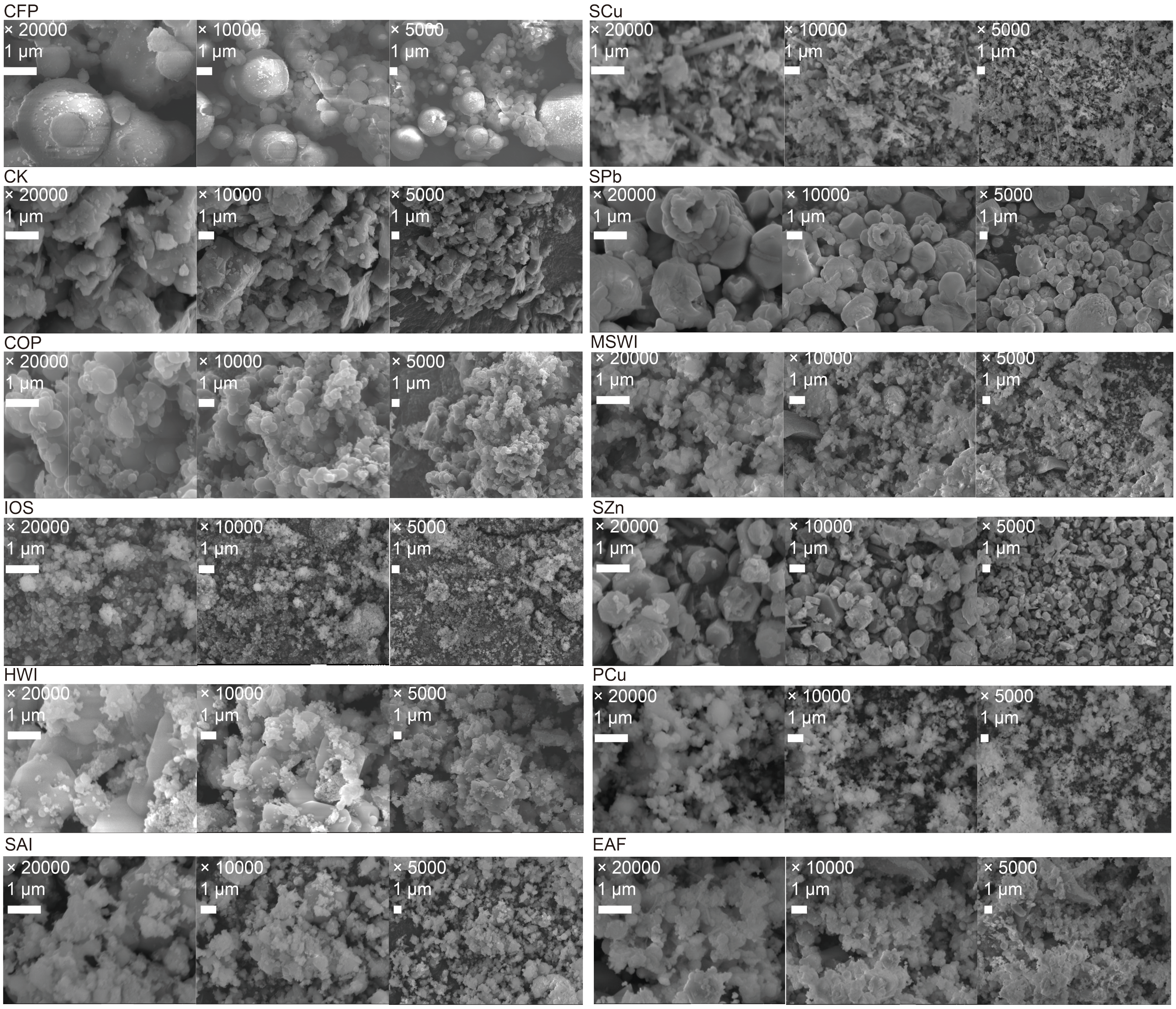

Supplementary Table 1

Information of polyhalogenated carbazole congeners detected in this research

Analytes	Abbreviation	CAS number	Molecular Formula	Structure
3-Chlorocarbazole	3-CCZ	2372-25-4	$C_{12}H_8ClN$	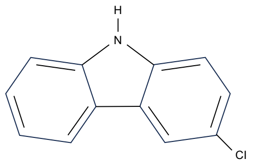
3,6-Dichlorocarbazole	3,6-CCZ	5599-71-3	$C_{12}H_7Cl_2N$	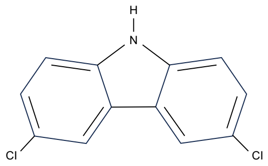
1,3,6,8-Tetrachlorocarbazole	1,3,6,8-CCZ	58910-96-6	$C_{12}H_5Cl_4N$	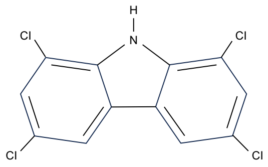
2,3,6,7-Tetrachlorocarbazole	2,3,6,7-CCZ	-	$C_{12}H_5Cl_4N$	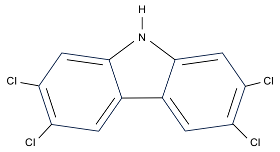
3-Bromocarbazole	3-BCZ	1592-95-6	$C_{12}H_8BrN$	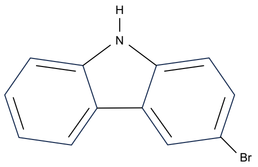
2,7-Dibromocarbazole	2,7-BCZ	136630-39-2	$C_{12}H_7Br_2N$	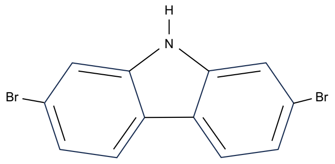
3,6-Dibromocarbazole	3,6-BCZ	6825-20-3	$C_{12}H_7Br_2N$	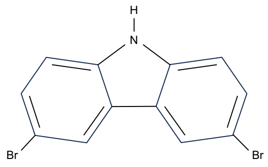
1,3,6-Tribromocarbazole	1,3,6-BCZ	55119-10-3	$C_{12}H_6Br_3N$	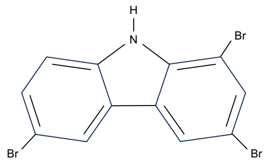
1,3,6,8-Tetrabromocarbazole	1,3,6,8-BCZ	55119-09-0	$C_{12}H_5Br_4N$	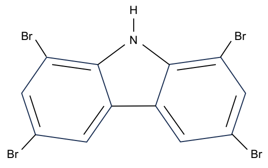
1-Bromo-3,6-Dichlorocarbazole	1-B-3,6-CCZ	100125-05-1	$C_{12}H_6BrCl_2N$	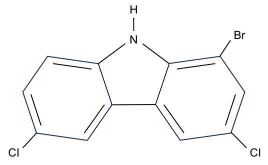
1,8-Dibromo-3,6-Dichlorocarbazole	1,8-B-3,6-CCZ	100131-03-1	$C_{12}H_5Br_2Cl_2N$	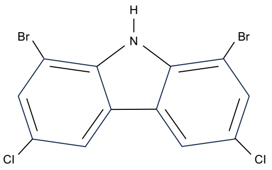

Supplementary Table 2

Information of samples collected in this research: total concentration of 11 polyhalogenated carbazoles (∑11PHCZ) and counterpart toxic equivalent quantities (TEQs)

Industry	Plants	Sample type	Raw materials	∑11PHCZs (pg/g)	Trimmean of ∑PHCZs (pg/g)	TEQs (pg/g)
Coke production	COP-1	i-PM[a]	Coking Coal	49402	30570	1.705
	COP-2	i-PM		4433		0.409
	COP-3	i-PM		85259		1.957
	COP-4	i-PM		149		0.008
	COP-5	i-PM		59050		1.297
	COP-6	i-PM		372		0.021
	COP-7	i-PM		108102		2.766
	COP-8	i-PM		15		0.000
	COP-9	i-PM		15		0.000
	COP-10	i-PM		187981		3.881
	COP-11	i-PM		10572		0.598
	COP-12	i-PM		12543		0.380
	COP-13	i-PM		36928		0.819
Coal-fired power	CFP-1	i-PM	coal	20	29	0.004
	CFP-2	i-PM		22		0.001
	CFP-3	i-PM		40		0.002
	CFP-4	i-PM		154		0.003
	CFP-5	i-PM		0		0.000
	CFP-6	i-PM		137		0.005
	CFP-7	i-PM		52		0.006
	CFP-8	i-PM		101		0.009
	CFP-9	i-PM		46		0.003
	CFP-10	i-PM		38		0.002
	CFP-11	i-PM		39		0.006
	CFP-12	i-PM		22		0.001
	CFP-13	i-PM		0		0.000
	CFP-14	i-PM		40		0.002
	CFP-15	i-PM		31		0.002
Electric arc furnace steel	EAF-1	i-PM	Scrap Steel, Pig Iron, Iron Alloy, Slagging Agent	377	1640	0.043
	EAF-2	i-PM		3223		1.387
	EAF-3	i-PM		2049		0.266
	EAF-4	i-PM		63		0.011
	EAF-5	i-PM		1390		0.133
	EAF-6	i-PM		136		0.008
	EAF-7	i-PM		2557		0.228
	EAF-8	i-PM		1424		0.067
	EAF-9	i-PM		3083		1.250
	EAF-10	i-PM		1136		0.581
	EAF-11	i-PM		438		0.018
	EAF-12	i-PM		2215		1.124
	EAF-13	i-PM		3038		1.383
	EAF-14	i-PM		1823		0.176
Co-processing in cement kin	CK-1	i-PM	laboratory waste	3781	390	0.903
	CK-2	i-PM	industrial waste	611		0.063
	CK-3	i-PM		766		0.022
	CK-4	i-PM		494		0.034

	CK-5	i-PM		8		0.000
	CK-6	i-PM		75		0.002
	MSWI-1	i-PM		154		0.026
	MSWI-2	i-PM		647		0.078
	MSWI-3	i-PM		28966		2.227
	MSWI-4	i-PM		1194		0.062
	MSWI-5	i-PM		402		0.021
	MSWI-6	i-PM		1516		0.071
Municipal solid waste incineration	MSWI-7	i-PM	municipal solid waste	3771	1077	1.650
	MSWI-8	i-PM		3058		0.255
	MSWI-9	i-PM		147		0.005
	MSWI-10	i-PM		1160		0.040
	MSWI-11	i-PM		57		0.002
	MSWI-12	i-PM		792		0.055
	MSWI-13	i-PM		27		0.000
	HWI-1	i-PM		0		0.000
	HWI-2	i-PM	–	207		0.025
	HWI-3	i-PM		14415		1.822
	HWI-4	i-PM		3099		0.430
	HWI-5	i-PM		356		0.037
Hazardous waste incineration	HWI-6	i-PM		193	601	0.127
	HWI-7	i-PM		6807		0.275
	HWI-8	i-PM	medical waste	183		0.045
	HWI-9	i-PM		482		0.128
	HWI-10	i-PM		313		0.147
	HWI-11	i-PM		242		0.098
	HWI-12	i-PM		936		0.201
	PCu-1	i-PM		223		0.021
	PCu-2	i-PM		367		0.040
	PCu-3	i-PM		82		0.003
Primary copper smelting	PCu-4	i-PM	primary copper	136	204	0.012
	PCu-5	i-PM		15		0.001
	PCu-6	i-PM		266		0.031
	PCu-7	i-PM		337		0.038
	SAl-1	i-PM		45122		8.152
	SAl-2	i-PM		2782		0.121
Secondary aluminum smelting	SAl-3	i-PM	aluminum scrap	474	2486	0.013
	SAl-4	i-PM		6569		0.146
	SAl-5	i-PM		210		0.021
	SAl-6	i-PM		2396		0.051

	SCu-1	i-PM		4550		0.507
	SCu-2	i-PM		3668		1.106
	SCu-3	i-PM		5624		1.320
Secondary copper smelting	SCu-4	i-PM	copper scrap	459	9173	0.086
	SCu-5	i-PM		553		0.183
	SCu-6	i-PM		15809		2.458
	SCu-7	i-PM		33547		14.665
	SZn-1	i-PM		8639		0.669
	SZn-2	i-PM		622		0.045
	SZn-3	i-PM		50		0.002
Secondary zinc smelting	SZn-4	i-PM	zinc scrap	51	675	0.001
	SZn-5	i-PM		407		0.063
	SZn-6	i-PM		1324		0.197
	SZn-7	i-PM		90		0.004
	SZn-8	i-PM		2178		0.385
	SPb-1	i-PM		22		0.004
	SPb-2	i-PM		0		0.000
	SPb-3	i-PM		0		0.000
	SPb-4	i-PM		0		0.000
	SPb-5	i-PM		0		0.000
Secondary lead smelting	SPb-6	i-PM	lead scrap	23	16	0.002
	SPb-7	i-PM		31		0.001
	SPb-8	i-PM		2962		0.128
	SPb-9	i-PM		15		0.000
	SPb-10	i-PM		0		0.000
	SPb-11	i-PM		68		0.002
	IOS-1	i-PM		60416		2.287
	IOS-2	i-PM		8324		0.312
	IOS-3	i-PM		6343		0.285
Iron ore sintering	IOS-4	i-PM	iron ore, lime, coke powder, pulverized coal	32076	19420	1.129
	IOS-5	i-PM		18787		0.697
	IOS-6	i-PM		7765		0.286
	IOS-7	i-PM		16984		0.588
	IOS-8	i-PM		4662		0.159
Organic chemical production	OC-1	bottom liquid	dichloroethane/chlorine	101781[b]	-	4.894
	OC-2	bottom liquid	benzene/chlorine	190691[b]	-	12.636

a: the abbreviation of industrial fine particulate matters

b: the unit is pg/mL

Supplementary Table 3

No-target screening of OC-1 using quadrupole-time of flight mass spectrometry

Name	CAS	Match sore	Formula
Benzene, 1,2-dichloro-	95-50-1	90.2	$C_6H_4Cl_2$
Benzene, 1,3-dichloro-	541-73-1	94	$C_6H_4Cl_2$
Cyclohexene, .gamma.-3,4,5,6-tetrachloro-	319-81-3	79.5	$C_6H_6Cl_4$
Benzene, 1,4-dichloro-	106-46-7	95	$C_6H_4Cl_2$
1,1'-Biphenyl, 4-chloro-	2051-62-9	98.5	$C_{12}H_9Cl$
1,1'-Biphenyl, 4-chloro-	2051-62-9	98.7	$C_{12}H_9Cl$
1,1'-Biphenyl, 3,3'-dichloro-	2050-67-1	97.1	$C_{12}H_8Cl_2$
1,1'-Biphenyl, 3,3'-dichloro-	2050-67-1	97.5	$C_{12}H_8Cl_2$
Benzene, 1,2,3-trichloro-	87-61-6	98.8	$C_6H_3Cl_3$
m-Terphenyl	92-06-8	98.7	$C_{18}H_{14}$
1,1'-Biphenyl, 2,2'-dichloro-	13029-08-8	97.1	$C_{12}H_8Cl_2$
Benzene, 1-chloro-4-methyl-	106-43-4	97.9	C_7H_7Cl
.delta.-Pentachlorocyclohexene	643-15-2	93	$C_6H_5Cl_5$
Phenol, 2-chloro-	95-57-8	97.8	C_6H_5ClO
.delta.-Pentachlorocyclohexene	643-15-2	94.3	$C_6H_5Cl_5$
1,1'-Biphenyl, 3,3'-dichloro-	2050-67-1	97.8	$C_{12}H_8Cl_2$
.alpha.-Lindane	319-84-6	96.7	$C_6H_6Cl_6$
Biphenyl	92-52-4	98.2	$C_{12}H_{10}$
Benzene, 1-bromo-4-chloro-	106-39-8	98.7	C_6H_4BrCl
m-Terphenyl	92-06-8	97.9	$C_{18}H_{14}$
1,1'-Biphenyl, 2-methyl-	643-58-3	97.4	$C_{13}H_{12}$
1,1'-Biphenyl, 3,3'-dichloro-	2050-67-1	96.8	$C_{12}H_8Cl_2$
Cyclopentane, (trichloroethenyl)-	55255-41-9	75	$C_7H_9Cl_3$
1,1'-Biphenyl, 4-chloro-	2051-62-9	96.7	$C_{12}H_9Cl$
p-Terphenyl, 2,5-dichloro-	61576-83-8	92.5	$C_{18}H_{12}Cl_2$
1,1'-Biphenyl, 4-methyl-	644-08-6	97.4	$C_{13}H_{12}$
5,5,10,10-Tetrachlorotricyclo[7.1.0.0(4,6)]decane	17725-81-4	61.9	$C_{10}H_{12}Cl_4$
1-Heptene, 5,7,7,7-tetrachloro-	51287-99-1	57.3	$C_7H_{10}Cl_4$
p-Terphenyl, 4-chloro-	1762-83-0	77.1	$C_{18}H_{13}Cl$
.alpha.-Lindane	319-84-6	92	$C_6H_6Cl_6$
1,1'-Biphenyl, 3,3'-dichloro-	2050-67-1	95.9	$C_{12}H_8Cl_2$
3-Chlorodiphenylmethane	27798-38-5	95.5	$C_{13}H_{11}Cl$
Benzene, nitro-	98-95-3	92.5	$C_6H_5NO_2$
Pyridazin-3(2H)-one, 5-chloro-2-methyl-4-(2-methylpropylamino)-	98795-97-2	70.8	$C_9H_{14}ClN_3O$

Supplementary Table 4

Concentrations of polyhalogenated carbazoles in industrial fine particulate matter from diverse industries

[a] not detected

Sample	Concentrations of each congener (pg/g)										
	3-CCZ	3-BCZ	3,6-CCZ	1,3,6,8-CCZ	1-B-3,6-CCZ	2,7-BCZ	3,6-BCZ	1,8-B-3,6-CCZ	1,3,6-BCZ	1,3,6,8-BCZ	2,3,6,7-CCZ
CFP-1	8	7	n.d.[a]	5	n.d.	n.d.	n.d.	n.d.	n.d.	n.d.	n.d.
CPF-2	8	7	7	n.d.	n.d.	n.d.	n.d.	n.d.	n.d.	n.d.	n.d.
CFP-3	8	16	7	n.d.	n.d.	n.d.	n.d.	n.d.	n.d.	n.d.	9
CFP-4	n.d.	154	n.d.	n.d.	n.d.	n.d.	n.d.	n.d.	n.d.	n.d.	n.d.
CFP-5	n.d.	n.d.	n.d.	n.d.	n.d.	n.d.	n.d.	n.d.	n.d.	n.d.	n.d.
CFP-6	8	108	22	n.d.	n.d.	n.d.	n.d.	n.d.	n.d.	n.d.	n.d.
CFP-7	8	23	7	5	n.d.	n.d.	n.d.	n.d.	n.d.	n.d.	9
CFP-8	8	17	14	5	n.d.	12	7	n.d.	n.d.	n.d.	38
CFP-9	8	7	15	n.d.	n.d.	n.d.	7	n.d.	n.d.	n.d.	9
CFP-10	17	10	10	n.d.	n.d.	n.d.	n.d.	n.d.	n.d.	n.d.	n.d.
CFP-11	8	7	19	5	n.d.	n.d.	n.d.	n.d.	n.d.	n.d.	n.d.
CFP-12	8	7	7	n.d.	n.d.	n.d.	n.d.	n.d.	n.d.	n.d.	n.d.
CFP-13	n.d.	n.d.	n.d.	n.d.	n.d.	n.d.	n.d.	n.d.	n.d.	n.d.	n.d.
CFP-14	8	16	7	n.d.	n.d.	n.d.	n.d.	n.d.	n.d.	n.d.	9
CFP-15	8	7	7	n.d.	n.d.	n.d.	n.d.	n.d.	n.d.	n.d.	9
CK-1	534	378	1538	1004	95	12	39	57	61	65	n.d.
CK-2	46	7	505	5	n.d.	12	7	n.d.	n.d.	n.d.	26
CK-3	256	446	64	n.d.	n.d.	n.d.	n.d.	n.d.	n.d.	n.d.	n.d.
CK-4	184	45	258	n.d.	n.d.	n.d.	7	n.d.	n.d.	n.d.	n.d.
CK-5	8	n.d.	n.d.	n.d.	n.d.	n.d.	n.d.	n.d.	n.d.	n.d.	n.d.
CK-6	75	n.d.	n.d.	n.d.	n.d.	n.d.	n.d.	n.d.	n.d.	n.d.	n.d.
COP-1	595	n.d.	8792	19	n.d.	15111	23712	n.d.	713	n.d.	459
COP-2	21	351	3416	n.d.	n.d.	195	243	n.d.	112	n.d.	95
COP-3	18983	60365	2466	52	n.d.	1420	1973	n.d.	n.d.	n.d.	n.d.
COP-4	29	39	52	n.d.	n.d.	12	7	n.d.	n.d.	n.d.	9
COP-5	9690	42574	1750	n.d.	n.d.	2311	2724	n.d.	n.d.	n.d.	n.d.
COP-6	90	106	143	n.d.	n.d.	12	21	n.d.	n.d.	n.d.	n.d.
COP-7	31182	70840	4102	260	n.d.	663	1055	n.d.	n.d.	n.d.	n.d.
COP-8	8	7	n.d.	n.d.	n.d.	n.d.	n.d.	n.d.	n.d.	n.d.	n.d.
COP-9	8	7	n.d.	n.d.	n.d.	n.d.	n.d.	n.d.	n.d.	n.d.	n.d.
COP-10	13476	163583	4256	5	n.d.	3088	3564	n.d.	n.d.	n.d.	9
COP-11	556	5411	1027	125	n.d.	352	327	11	202	11	2548
COP-12	5846	5577	1101	n.d.	n.d.	12	7	n.d.	n.d.	n.d.	n.d.
COP-13	2544	29142	1533	n.d.	n.d.	1576	2123	n.d.	n.d.	n.d.	9
EAF-1	8	7	348	5	n.d.	n.d.	n.d.	n.d.	n.d.	n.d.	9
EAF-2	79	100	430	1850	n.d.	161	137	291	48	n.d.	128
EAF-3	114	234	97	250	128	236	367	25	119	n.d.	479
EAF-4	8	7	7	13	n.d.	12	7	n.d.	n.d.	n.d.	9
EAF-5	523	435	207	127	7	25	35	n.d.	8	n.d.	23
EAF-6	66	21	16	5	n.d.	12	7	n.d.	n.d.	n.d.	9
EAF-7	1304	716	126	240	5	63	75	11	8	n.d.	9
EAF-8	368	708	255	10	2	12	31	11	8	11	9
EAF-9	167	306	189	1616	n.d.	109	235	461	n.d.	n.d.	n.d.

EAF-10	41	71	108	844	29	12	n.d.	11	8	11	n.d.
EAF-11	244	107	87	n.d.	n.d.	n.d.	n.d.	n.d.	n.d.	n.d.	n.d.
EAF-12	n.d.	n.d.	613	1601	n.d.	n.d.	n.d.	n.d.	n.d.	n.d.	n.d.
EAF-13	n.d.	104	213	1838	n.d.	184	269	430	n.d.	n.d.	n.d.
EAF-14	111	148	19	34	n.d.	27	25	n.d.	66	n.d.	1394
HWI-1	n.d.	n.d.	n.d.	n.d.	n.d.	n.d.	n.d.	n.d.	n.d.	n.d.	n.d.
HWI-2	n.d.	99	n.d.	32	n.d.	34	32	n.d.	n.d.	n.d.	9
HWI-3	8	56	13525	496	n.d.	101	229	n.d.	n.d.	n.d.	n.d.
HWI-4	1532	263	836	440	n.d.	12	7	n.d.	n.d.	n.d.	9
HWI-5	42	n.d.	290	5	n.d.	12	7	n.d.	n.d.	n.d.	n.d.
HWI-6	n.d.	n.d.	n.d.	193	n.d.	n.d.	n.d.	n.d.	n.d.	n.d.	n.d.
HWI-7	411	4353	599	150	n.d.	461	832	n.d.	n.d.	n.d.	n.d.
HWI-8	40	38	49	57	n.d.	n.d.	n.d.	n.d.	n.d.	n.d.	n.d.
HWI-9	24	n.d.	318	140	n.d.	n.d.	n.d.	n.d.	n.d.	n.d.	n.d.
HWI-10	52	7	41	213	n.d.	n.d.	n.d.	n.d.	n.d.	n.d.	n.d.
HWI-11	30	13	61	137	n.d.	n.d.	n.d.	n.d.	n.d.	n.d.	n.d.
HWI-12	90	561	n.d.	285	n.d.	n.d.	n.d.	n.d.	n.d.	n.d.	n.d.
PCu-1	8	7	165	n.d.	n.d.	12	7	n.d.	n.d.	n.d.	24
PCu-2	8	7	319	5	n.d.	12	7	n.d.	n.d.	n.d.	9
PCu-3	75	n.d.	7	n.d.	n.d.	n.d.	n.d.	n.d.	n.d.	n.d.	n.d.
PCu-4	36	n.d.	32	5	n.d.	12	7	n.d.	n.d.	n.d.	44
PCu-5	8	n.d.	7	n.d.	n.d.	n.d.	n.d.	n.d.	n.d.	n.d.	n.d.
PCu-6	8	7	237	5	n.d.	n.d.	n.d.	n.d.	n.d.	n.d.	9
PCu-7	8	7	308	5	n.d.	n.d.	n.d.	n.d.	n.d.	n.d.	9
SAl-1	69	66	1564	8752	857	8766	10223	1800	2741	51	10234
SAl-2	587	896	212	31	n.d.	308	400	n.d.	8	n.d.	341
SAl-3	460	7	7	n.d.	n.d.	n.d.	n.d.	n.d.	n.d.	n.d.	n.d.
SAl-4	404	4056	168	16	n.d.	797	1076	n.d.	51	n.d.	n.d.
SAl-5	8	7	7	n.d.	n.d.	12	7	11	n.d.	n.d.	158
SAl-6	854	1516	7	n.d.	n.d.	12	7	n.d.	n.d.	n.d.	n.d.
SCu-1	208	69	164	166	45	105	126	n.d.	41	n.d.	3627
SCu-2	90	433	736	1396	n.d.	80	69	25	34	n.d.	806
SCu-3	1518	532	674	1702	n.d.	420	303	68	147	n.d.	259
SCu-4	269	32	47	110	n.d.	n.d.	n.d.	n.d.	n.d.	n.d.	n.d.
SCu-5	43	43	24	261	n.d.	33	116	n.d.	n.d.	n.d.	32
SCu-6	3664	1054	6270	2086	7	n.d.	n.d.	n.d.	n.d.	n.d.	2728
SCu-7	4940	7	7900	20700	n.d.	n.d.	n.d.	n.d.	n.d.	n.d.	n.d.
SPb-1	8	n.d.	n.d.	5	n.d.	n.d.	n.d.	n.d.	n.d.	n.d.	9
SPb-2	n.d.	n.d.	n.d.	n.d.	n.d.	n.d.	n.d.	n.d.	n.d.	n.d.	n.d.
SPb-3	n.d.	n.d.	n.d.	n.d.	n.d.	n.d.	n.d.	n.d.	n.d.	n.d.	n.d.
SPb-4	n.d.	n.d.	n.d.	n.d.	n.d.	n.d.	n.d.	n.d.	n.d.	n.d.	n.d.
SPb-5	n.d.	n.d.	n.d.	n.d.	n.d.	n.d.	n.d.	n.d.	n.d.	n.d.	n.d.
SPb-6	8	n.d.	15	n.d.	n.d.	n.d.	n.d.	n.d.	n.d.	n.d.	n.d.
SPb-7	8	23	n.d.	n.d.	n.d.	n.d.	n.d.	n.d.	n.d.	n.d.	n.d.
SPb-8	1748	416	611	5	n.d.	97	84	n.d.	n.d.	n.d.	n.d.
SPb-9	8	7	n.d.	n.d.	n.d.	n.d.	n.d.	n.d.	n.d.	n.d.	n.d.
SPb-10	n.d.	n.d.	n.d.	n.d.	n.d.	n.d.	n.d.	n.d.	n.d.	n.d.	n.d.
SPb-11	68	n.d.	n.d.	n.d.	n.d.	n.d.	n.d.	n.d.	n.d.	n.d.	n.d.
SZn-1	3057	301	3515	36	n.d.	12	19	n.d.	n.d.	n.d.	1699
SZn-2	282	n.d.	95	5	n.d.	n.d.	n.d.	n.d.	n.d.	n.d.	240
SZn-3	8	7	7	n.d.	n.d.	12	7	n.d.	n.d.	n.d.	9
SZn-4	31	20	n.d.	n.d.	n.d.	n.d.	n.d.	n.d.	n.d.	n.d.	n.d.
SZn-5	186	20	28	77	26	23	37	n.d.	n.d.	n.d.	9

SZn-6	633	132	309	216	n.d.	26	n.d.	n.d.	n.d.	n.d.	9
SZn-7	59	15	7	n.d.	n.d.	n.d.	n.d.	n.d.	n.d.	n.d.	9
SZn-8	679	n.d.	1124	366	n.d.	n.d.	n.d.	n.d.	n.d.	n.d.	9
MSWI-1	18	n.d.	41	22	n.d.	n.d.	7	n.d.	n.d.	n.d.	66
MSWI-2	47	72	232	39	n.d.	12	61	11	37	11	125
MSWI-3	11767	751	12607	653	291	1135	1600	11	33	n.d.	118
MSWI-4	317	376	114	n.d.	n.d.	28	26	n.d.	n.d.	n.d.	334
MSWI-5	40	221	140	n.d.	n.d.	n.d.	n.d.	n.d.	n.d.	n.d.	n.d.
MSWI-6	210	850	456	n.d.	n.d.	n.d.	n.d.	n.d.	n.d.	n.d.	n.d.
MSWI-7	22	497	592	2362	196	39	53	11	n.d.	n.d.	n.d.
MSWI-8	87	84	32	n.d.	50	150	235	11	553	n.d.	1855
MSWI-9	8	97	30	n.d.	n.d.	12	n.d.	n.d.	n.d.	n.d.	n.d.
MSWI-10	440	477	161	n.d.	n.d.	12	70	n.d.	n.d.	n.d.	n.d.
MSWI-11	57	n.d.	n.d.	n.d.	n.d.	n.d.	n.d.	n.d.	n.d.	n.d.	n.d.
MSWI-12	280	360	66	50	n.d.	12	24	n.d.	n.d.	n.d.	n.d.
MSWI-13	8	n.d.	n.d.	n.d.	n.d.	12	7	n.d.	n.d.	n.d.	n.d.
IOS-1	19569	28052	11055	17	n.d.	743	980	n.d.	n.d.	n.d.	n.d.
IOS-2	3507	2360	1452	n.d.	n.d.	380	625	n.d.	n.d.	n.d.	n.d.
IOS-3	1946	1499	1696	n.d.	n.d.	458	744	n.d.	n.d.	n.d.	n.d.
IOS-4	17465	8779	4333	n.d.	n.d.	563	936	n.d.	n.d.	n.d.	n.d.
IOS-5	9303	4355	3017	5	n.d.	797	1311	n.d.	n.d.	n.d.	n.d.
IOS-6	3517	2027	1274	n.d.	n.d.	418	529	n.d.	n.d.	n.d.	n.d.
IOS-7	8257	4210	2328	n.d.	n.d.	952	1239	n.d.	n.d.	n.d.	n.d.
IOS-8	2480	773	593	n.d.	n.d.	261	555	n.d.	n.d.	n.d.	n.d.

Supplementary Table 5

Particulate matter emission factors (EFs) of diverse production scales, raw materials, end products, and procedures[15]

a: CL(T): Coal loading (Top-charging) b: CD(T): Coke Discharging (Top-charging) c: BFGH(T): Blast Furnace Gas Heating (Top-charging) d: DCQ(T): Dry Coke Quenching (Top-charging) e: CL(S): Coal Loading (Stamp-Charging) f: CD(S): Coke Discharging (Stamp-Charging) g: BFGH(S): Blast Furnace Gas Heating (Stamp-Charging) h: WCQ(S): Wet Coke Quenching (Stamp-Charging) i: PMEFs: Emission Factor of PM

Industry	Production Process	Production Scale	Raw Material	Product	PM Producing Coefficient (kg/t)	PM removal equipment	PM removal efficiency	Final PM EFs (g/t)	PMEFs[i] for Estimation (g/t)
SCu	Refining	-	Crude Copper	Anode Copper	0.07	Bag Filter	99%	1	479
	Fire Melting	-	Low-grade Copper Scrap	Crude Copper	31.07	Bag Filter	98%	621	
	Pyro-refining	-	How-grade Copper Scrap	Anode Copper	16.72	Bag Filter	98%	334	
	electrolyzation	-	Anode Copper	Cathode Copper	-	Bag Filter	-	-	
COP	CL(T) [a]	Owen Height > 6 m	Coking Coal	Coke	-	Bag Filter	-	102	442
	CD(T) [b]				-	Bag Filter	-	129	
	BFGH(T) [c]				-	Bag Filter	-	114	
	DCQ(T) [d]				-	Bag Filter	-	105	
	CL(T)	6m > Owen Height > 4.3 m			-	Bag Filter	-	121	
	CD(T)				-	Bag Filter	-	134	
	BFGH(T)				-	Bag Filter	-	119	
	DCQ(T)				-	Bag Filter	-	113	
	CL(T)	4.3m > Owen Height			-	Bag Filter	-	174	
	CD(T)				-	Bag Filter	-	120	
	BFGH(T)				-	Bag Filter	-	142	
	DCQ(T)				-	Bag Filter	-	124	
	CL(S)[e]	-			-	Bag Filter	-	115	
	CD(S)[f]				-	Bag Filter	-	131	
	BFGH(S)[g]				-	Bag Filter	-	120	
	WCQ(S)h				-	Bag Filter	-	68	
EAF	Electric Furnace Steelmaking	-	Scrap Steel, Pig Iron, Iron Alloy, Slagging Agent	Carbon Steel	26.19	Bag Filter	99.60%	105	105
		> 50 t	Scrap Steel, Hot Iron, Ferrochrome, Direct Reduced Iron, Slagging Agent	Alloy Steel	13.15	Bag Filter	99.60%	53	
		< 50 t			17.99	Bag Filter	99.60%	72	

		-	Scrap Steel, Hot Iron, Ferrochrome, Direct Reduced Iron, Slagging Agent	Stainless Steel	78.4	Bag Filter	99.60%	314	
		sintering machines > 360 m^2			5.76	Bag Filter	0.9957	25	
	Sintering Machine Head	180 m^2 < sintering machines < 360 m^2			5.81	Bag Filter	0.9957	25	
		sintering machines < 180 m^2			3.45	Bag Filter	0.9957	15	
		sintering machines > 360 m^2			5.96	Bag Filter	0.9957	26	
IOS	Sintering Machine Tail	180 m^2 < sintering machines < 360 m^2	Iron ore, Lime, Coke Powder, Pulverized Coal	Sinter	4.88	Bag Filter	0.9957	21	65
		sintering machines < 180 m^2			3.6	Bag Filter	0.9957	15	
		sintering machines > 360 m^2			6.54	Bag Filter	0.9957	28	
	Discharge	180 m^2 < sintering machines < 360 m^2			5.6	Bag Filter	0.9957	24	
		sintering machines < 180 m^2			3.75	Bag Filter	0.9957	16	

Supplementary Table 6

The relative effect potencies (REPs) of polyhalogenated carbazoles[16]

Congeners	Range of REPs	
	CYP1A1	CYP1B1
2,3,7,8-Tetrachlorodibenzodioxin	1	1
3-Chlorocarbazole	2.7×10^{-5}	-
3,6-Dichlorocarbazole	1.1×10^{-4}	-
1,3,6,8-Tetrachlorocarbazole	6.6×10^{-4}	5.8×10^{-3}
2,3,6,7-Tetrachlorocarbazole	1.0×10^{-4}	3.2×10^{-3}
3-Bromocarbazole	1.8×10^{-5}	2.6×10^{-4}
2,7-Dibromocarbazole	1.3×10^{-5}	1.3×10^{-4}
3,6-Dibromocarbazole	1.7×10^{-5}	1.5×10^{-4}
1,3,6-Tribromocarbazole	9.0×10^{-5}	8.5×10^{-4}
1,3,6,8-Tetrabromocarbazole	3.1×10^{-4}	9.7×10^{-3}
1-Bromo-3,6-Dichlorocarbazole	6.0×10^{-5}	3.3×10^{-4}
1,8-Dibromo-3,6-Dichlorocarbazole	3.2×10^{-4}	9.7×10^{-3}

Supplementary Method 1

Data processing method for characteristics of polyhalogenated carbazole (PHCZ) concentrations, congener profile analysis, principal component analysis, and emission estimation

Characteristics of PHCZ concentrations: For industrial sources, the average concentration of each congener in i-PM samples from all factories in a specific industry is used for the calculation of Spearman's coefficients among 11 congeners in i-PM samples. For environmental occurrences, the average concentration of each congener in PHCZ investigation of soil or sediment is used for the calculation of Spearman's coefficients among 11 congeners in environmental media. Data analysis is conducted using SPSS.

Congener profile analysis: For industrial sources, the average proportion of each congener in i-PM samples from all factories in the specific industry is used to represent the PHCZ congener profile of this industry. For environmental occurrences, the average proportion of each congener in PHCZ investigation of soil or sediment is used to represent the PHCZ congener profile in this environmental matrix.

Principal component analysis: principal component analysis is conducted for 120 i-PM samples and 16 environmental occurrences based on individual congener profile of PHCZs. Data analysis is conducted using SPSS.

Emission estimation: To reduce the variance among total PHCZ concentrations of i-PM samples in each industry, minimaxes are removed through the box plot, which is located at ±1.5 inter-quartile range (IQR). The remaining PHCZ concentrations are averaged to obtain the PHCZ content of i-PM samples from each industry.

Supplementary Method 2

Sample pretreatment and instrumental analysis[17]

0.03 mL bottom liquid and 5 g industrial particulate matters are used for detection of PHCZ concentration. The clean-up standard ^{13}C-1,3,6,8-MCCZ was added at 1 μg/mL to give 10 ng in each sample. All samples were left for 24 h for aging before pretreatment. 5g i-PM were Soxhlet extracted with acetone/n-hexane (1:1, v/v) for 24 h and then evaporated to 1-2 mL. For purification, the extracts of i-PM and 0.03 mL bottom liquid were added to silica gel columns and eluted with 100 mL of n-hexane/dichloromethane (4:1, v/v). The columns were packed from bottom to top with 8 g of silica gel and 4 g of anhydrous granulated sodium sulfate. The silica gel was baked at 450 °C for 6 h to remove any residues before use. Each eluate was concentrated to approximately 40 μL, first by rotatory evaporation and then by drying under a nitrogen blower. The injection standard ^{13}C-3,6-MCCZ (1 ng/mL, 10 ng) was added to each concentrated sample.

Analysis of target compounds was performed using a model 7890A gas chromatograph coupled to a model 7010 triple quadrupole mass spectrometer (both from Agilent Technologies, Santa Clara, CA, USA) equipped with a multimode inlet. Separation was conducted on a DB-5 MS Ultra inert capillary column (30 m × 0.25 mm i.d., 0.25 μm film thickness; Agilent Technologies). Helium at a constant flow rate of 1.3 mL/min was used as the carrier gas. The oven temperature was initially set at 70 °C, held at 70 °C for 3 min, and then increased to 230 °C at a rate of 20 °C /min. After holding at 230 °C for 3 min, a slow increase of 1 °C /min was used to separate 3,6-BCZ and 1,8-B-3,6-CCZ, which had similar retention times. After reaching 240 °C, the temperature was held for 3 min, before increasing to 300 °C at 20 °C /min. The injection volume was 2 μL and the samples were injected in pulsed spitless mode with an inlet temperature of 290 °C. The temperatures of the MS transfer line and high-sensitivity electron impact ionization source were set at 280 °C and 230 °C, respectively. Measurements were performed using multiple reaction monitoring (MRM) mode and positive electron impact ionization. The collision cell gases were nitrogen (1.5 mL/min) and helium (2.25 mL/min). The ions used for quantification and qualification are shown below.

GC/MS/MS parameters used in the determination of carbazoles

a: retention time (min)

b: collision energy (eV)

Compounds	molecular weight	RT[a]	Quantifier(m/z)	CE[b]	Quantifier(m/z)	CE
3-CCZ	201.7	13.22	201 > 166	20	203 > 166	20
3-BCZ	246.1	14.42	245 > 166	20	247 > 166	20
3,6-CCZ	236.1	16.41	235 > 200	20	200 > 164	20
^{13}C-3,6-CCZ	248.1	16.41	247 > 212	20	212 > 176	20
1,3,6,8-CCZ	305	17.02	305 > 270	18	303 > 268	15
^{13}C-1,3,6,8-CCZ	317	17.02	317 > 282	18	315 > 280	15
1-B-3,6-CCZ	315	18.71	315 > 164	35	313 > 234	20
2,7-BCZ	325	20.97	325 > 165	35	323 > 244	25
3,6-BCZ	325	21.23	325 > 165	35	323 > 244	25
1,8-B-3,6-CCZ	393.9	21.31	393 > 198	40	395 > 233	42
1,3,6-BCZ	403.9	25	403 > 164	47	405 > 245	40
1,3,6,8-BCZ	482.8	28.24	483 > 323	35	483 > 404	37
2,3,6,7-BCZ	305	28.86	305 > 270	30	305 > 198	40

Supplementary Method 3

Calculation method for toxic equivalent quantities (TEQs) of polyhalogenated carbazoles (PHCZs) in industrial fine particulate matters and chemical bottom liquid.

The TEQ is a widely used to estimate the potential toxic effects of many DLCs[18]. For PHCZs, Riddle et al.[16] determined the relative effect potencies (REPs) of PHCZs on the basis of structure-dependent induction of cytochrome P450 1A1 mRNAs in MDA-MB-468 breast cancer cells, making it feasible to determine the TEQs of the concentrations of PHCZs in each industry. The TEQ of the concentration (abbreviated as TEQ_C) of each sample is calculated using equation (1):

$$TEQ_C = \sum C_i \times REP_i \qquad (1)$$

where C_i and REP_i represent the concentration and REP of each congener in each sample, respectively.

Supplementary References:

1. Chen, W. L. *et al.* Quantitative determination of ultra-trace carbazoles in sediments in the coastal environment. *Chemosphere* **150**, 586–595 (2016).
2. Wu, Y., Tan, H., Sutton, R. & Chen, D. From Sediment to Top Predators: Broad Exposure of Polyhalogenated Carbazoles in San Francisco Bay (U.S.A.). *Environ. Sci. Technol.* **51**, 2038–2046 (2017).
3. Wu, Y., Qiu, Y., Tan, H. & Chen, D. Polyhalogenated carbazoles in sediments from Lake Tai (China): Distribution, congener composition, and toxic equivalent evaluation. *Environ. Pollut.* **220**, 142–149 (2017).
4. Zhou, Y. *et al.* Method development for analyzing ultratrace polyhalogenated carbazoles in soil and sediment. *Ecotoxicol. Environ. Saf.* **182**, 109470 (2019).
5. Jia, Y. *et al.* Sediment-water distribution and potential sources of polyhalogenated carbazoles in a coastal river locating at a north metropolis, China. *Mar. Pollut. Bull.* **189**, 114790 (2023).
6. Tao, W. *et al.* Determination of polyhalogenated carbazoles in soil using gas chromatography-triple quadrupole tandem mass spectrometry. *Sci. Total Environ.* **710**, (2020).
7. Liu, M. *et al.* Occurrence and potential sources of polyhalogenated carbazoles in farmland soils from the Three Northeast Provinces, China. *Sci. Total Environ.* **799**, 149459 (2021).
8. Liu, M. *et al.* Occurrence and distribution of polyhalogenated carbazoles in eastern Tibetan Plateau soils along the slope of Mt. Qionglai. *Chemosphere* **298**, 134200 (2022).
9. Su, Q. *et al.* Soil to earthworm bioaccumulation of polyhalogenated carbazoles and related compounds: Lab and field tests. *Environ. Pollut.* **316**, (2023).
10. Fromme, H. *et al.* Occurrence of carbazoles in dust and air samples from different locations in Germany. *Sci. Total Environ.* **610–611**, 412–418 (2018).
11. Zhou, H., Dong, X., Zhao, N., Zhao, M. & Jin, H. Polyhalogenated carbazoles in indoor dust from Hangzhou, China. *Sci. Total Environ.* **859**, 159971 (2023).
12. Ministry of Ecology and Environment of the People's Republic of China. Specification for Compilation of Emission Standards for Atmospheric Pollutants of the Coking Chemical Industry. https://www.mee.gov.cn (2021).
13. World Steel Association. World Steel in Figures 2020. https://www.worldsteel.org/mediacentre/press-releases/2019/World-Steel-in-Figures-2019.html. (2020).
14. the Word Bank. What-a-Waste-Global-Database. https://datacatalog.worldbank.org/search/dataset/0039597/What-a-Waste-Global-Database (2019).
15. Ministry of Ecology and Environment of the People's Republic of China. Handbook of Emission Sources Inventory Survey, Pollution Discharge Calculation Methods, and Coefficients. https://www.mee.gov.cn (2021).
16. Riddell, N. *et al.* Characterization and Biological Potency of Mono- to Tetra-Halogenated Carbazoles. *Environ. Sci. Technol.* **49**, 10658–10666 (2015).
17. Sun, Y. *et al.* Method development for determination of polyhalogenated carbazoles in industrial waste through gas chromatography/triple quadrupole tandem mass spectrometry. *Rapid Commun. Mass Spectrom.* **36**, 9324 (2022).
18. Van Den Berg, M. *et al.* Toxic equivalency factors (TEFs) for PCBs, PCDDs, PCDFs for humans and wildlife. *Environ. Health Perspect.* **106**, 775–792 (1998).
